# Amorphous Solid Dispersion as Drug Delivery Vehicles in Cancer

**DOI:** 10.3390/polym15163380

**Published:** 2023-08-11

**Authors:** Arif Budiman, Annisa Luthfiyah Handini, Mutia Nur Muslimah, Neng Vera Nurani, Eli Laelasari, Insan Sunan Kurniawansyah, Diah Lia Aulifa

**Affiliations:** 1 Department of Pharmaceutics and Pharmaceutical Technology, Faculty of Pharmacy, Universitas Padjadjaran, Jl. Raya Bandung-Sumedang Km. 21, Bandung 45363, Indonesia; annisa20022@mail.unpad.ac.id (A.L.H.); mutia20002@mail.unpad.ac.id (M.N.M.); neng19001@mail.unpad.ac.id (N.V.N.); eli19001@mail.unpad.ac.id (E.L.); insan.sunan.kurniawansyah@unpad.ac.id (I.S.K.); 2 Department of Pharmaceutical Analysis and Medicinal Chemistry, Faculty of Pharmacy, Universitas Padjadjaran, Jl. Raya Bandung-Sumedang Km. 21, Bandung 45363, Indonesia; diah.lia@unpad.ac.id

**Keywords:** amorphous solid dispersion, poorly water-soluble anticancer drugs, dissolution, bioavailability

## Abstract

Cancer treatment has improved over the past decades, but a major challenge lies in drug formulation, specifically for oral administration. Most anticancer drugs have poor water solubility which can affect their bioavailability. This causes suboptimal pharmacokinetic performance, resulting in limited efficacy and safety when administered orally. As a result, it is essential to develop a strategy to modify the solubility of anticancer drugs in oral formulations to improve their efficacy and safety. A promising approach that can be implemented is amorphous solid dispersion (ASD) which can enhance the aqueous solubility and bioavailability of poorly water-soluble drugs. The addition of a polymer can cause stability in the formulations and maintain a high supersaturation in bulk medium. Therefore, this study aimed to summarize and elucidate the mechanisms and impact of an amorphous solid dispersion system on cancer therapy. To gather relevant information, a comprehensive search was conducted using keywords such as “anticancer drug” and “amorphous solid dispersion” in the PubMed, Scopus, and Google Scholar databases. The review provides an overview and discussion of the issues related to the ASD system used to improve the bioavailability of anticancer drugs based on molecular pharmaceutics. A thorough understanding of anticancer drugs in this system at a molecular level is imperative for the rational design of the products.

## 1. Introduction

Cancer is a global health problem responsible for worldwide mortality [1]. Despite the increase in studies related to the discovery and development of anticancer drugs, the success rate of these medications has remained poor and needs improvement over existing options [2]. A significant factor contributing to the poor efficacy is inadequate pharmacokinetics, primarily caused by limited water solubility [3,4,5,6]. About 75% of newly developed drug candidates, including anticancer drugs, have poor water solubility [4]. Furthermore, around 65% of currently approved anticancer oral medicines also suffer from this limitation, restricting their potential therapeutic outcomes [7]. For example, Nexavar^®^ (sorafenib tosylate) and sorafenib are classified as BCS Class II according to the biopharmaceutical classification system (BCS), indicating low solubility and high permeability. Dissolution of sorafenib in the gastrointestinal tract occurs slowly, serving as a crucial factor that limits its absorption. This causes low oral bioavailability [8], thereby leading to either acute toxicity or sub-therapeutic outcomes [9,10] Paclitaxel has also poor water solubility (<0.03 mg/mL) [11], necessitating the addition of Cremophor EL (polyethoxylated castor oil) and ethanol. Despite these measures, patients still experience adverse reactions, some of which can be life-threatening [12,13]. Therefore, the challenge of improving the water solubility of existing anticancer drugs remains a significant hurdle, particularly concerning oral administration.

The amorphous system was a promising strategy in the formulations due to its ability to enhance the dissolution rate and bioavailability of poorly water-soluble drugs [14]. Amorphous drugs have higher free energy compared to their crystalline counterparts, resulting in improved aqueous solubility and maintenance of high supersaturation levels [15]. However, amorphous drugs are unstable and easy to recrystallize. Therefore, the addition of excipients such as polymers was necessary for a solid formulation to inhibit drug recrystallization [16,17].

Amorphous solid dispersion (ASD) is a formulation technology used to stabilize amorphous drugs. In this method, the drug is dispersed in a carrier, thereby lowering the total energy required for the solubilization [18]. Polymers as carriers also play a key role in the improvement of solubility and bioavailability through their interaction. They can stabilize the ASD system, prevent drug recrystallization through interaction between the drug–polymer, and improve physical stability under a variety of accelerated conditions, such as elevated temperature as well as relative humidity [19]. Therefore, ASD technology offers potential benefits for cancer treatment by enhancing the solubility of poorly water-soluble anticancer drugs such as regorafenib, vemurafenib, and others [7,20,21].

Several studies have highlighted the potential of ASD technology in increasing the water solubility of anticancer drugs. However, there is no detailed information about the mechanism by which these drugs interact within the ASD system, as well as the impact of the interactions on solubility improvement, pharmaceutical properties, and anticancer activity. Therefore, this study aimed to summarize and discuss the solubility improvement techniques employed in ASD systems for poorly water-soluble anticancer drugs. The results were expected to be more valuable, as the objectives were to explain the mechanisms of poorly water-soluble anticancer drugs and the effect of ASD on the solubility, dissolution, physical stability, and anticancer activity [22,23].

## 2. Anticancer Drugs

Cancer is a major public health issue and one of the main causes of death worldwide [1]. Oral administration of anticancer drugs is the most preferred method, as it has many benefits, including ease of use and lower cost of therapy. Approximately 65% of currently licensed oral anticancer drugs have poor water solubility, leading to suboptimal therapeutic outcomes with minimal efficacy and toxicity [7]. The limited water solubility can be attributed to two main factors. Firstly, during the discovery phase, insufficient attention is given to the physicochemical features of the candidates. Secondly, some important hydrophobic structural properties are required for the permeability, activity, and stability of these drugs, resulting in poor aqueous solubility.

A major limitation in the anticancer drug development pipeline has been a lack of focus on drug disposition and pharmacodynamics. The application of the “nanomolar rule” was prevalent in the development process. This criterion dictates the use of substances with nanomolar potency. Furthermore, it was discovered on the premise that such substances would be safe and effective in modest doses. An important factor such as the physicochemical features of these drugs, which influence their pharmacokinetics as well as safety and efficacy, was overlooked. Therefore, compounds with excellent cytotoxicity in nanomolar concentrations, such as combretastatin A-4, were selected for development, but ultimately proved unsuccessful due to their poor water solubility.

Anticancer medications need to possess a particular level of hydrophobicity or lipophilicity to penetrate cell membranes and reach their site of action. Veber et al. [24] stated that substances with a topological polar surface area (TPSA) of less than 140 have adequate permeability. However, the solubility of water decreases with reduced polar surface area. Striking a balance between the two conflicting elements of high solubility and low polarity was very challenging. Therefore, drug solubility is very important as it is related to bioavailability. Anticancer medications with poor solubility cause suboptimal pharmacokinetic performance. Additionally, they exhibit limited maximum effectiveness when the drug is administered orally.

The crystalline state of anticancer drugs also contributes to their poor water solubility. This form provides several advantages, including great purity and stability. However, the dissolution of the crystalline form requires overcoming the lattice energy barrier, which is difficult and leads to slower drug breakdown. Table 1 summarizes examples of poorly water-soluble anticancer drugs with the mechanism of their anticancer activity. The amorphous form of the anticancer drug would be more water soluble but more prone to physical instability [25]. Thus, developing a strategy to stabilize amorphous drugs is needed in the formulation of poorly water-soluble anticancer drugs.

## 3. Amorphous Solid Dispersion

**Figure 1:**
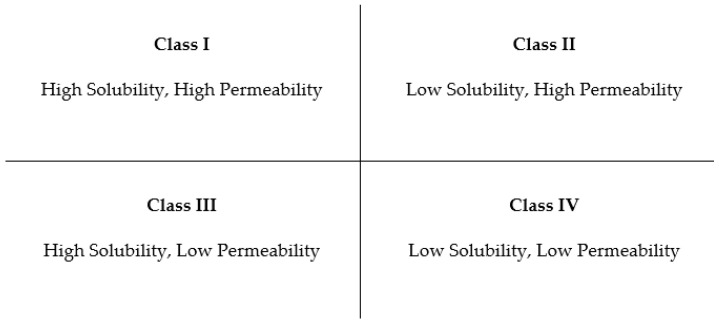
The classification of drugs based on solubility and permeability.

Solubility is one of the problems in drug formulation, particularly for oral dosage forms, which hardly dissolve in gastrointestinal fluids. Furthermore, it can be classified into four classes according to the Biopharmaceutical Classification System (BCS). Four classes of BCS show solubility and permeability, as shown in Figure 1. In class II, modifications such as solid dispersion, particle size reduction, and nanoparticles are employed to improve solubility [25].

Several methods were applied to increase solubility, and one of them was amorphous solid dispersions. The amorphous materials involved were thermodynamically metastable and could be changed into crystals that were in a more stable form [19]. Furthermore, they are often described as a glassy and supercooled liquid that can be reached by rapid cooling. When the material was cooled, the viscosity was increased and the molecular mobility was decreased simultaneously. The process was called temperature glass transition and the material was known as glass [67]. Similar to the amorphous form, the glass which was a brittle solid with no crystalline structure was also metastable and related to the crystalline form of the drug. The transition was needed because sudden cooling (under the glass transition temperature) increases the entropy of the amorphous crystal, leading to higher enthalpy, entropy, and free energy. A mathematical equation was used to predict an increase in solubility. However, the value from the experimental results may be lower. The amorphous form possessed higher solubility due to its greater free energy [25].

The drug in an amorphous form can be transformed into an amorphous solid dispersion, thereby improving its bioavailability. In this process, the polymer carrier played an important role by increasing the dissolution rate, enhancing drug solubility, and improving the physical stability of the solid state. Additionally, it can reduce molecular mobility and raise the glass transition temperature, resulting in increased stability during the conversion from a crystalline into amorphous form. The drug–polymer interaction contributed to the stabilization by disrupting the intermolecular interaction between the drug crystal lattice. The presence of the polymer alters the nature of the crystal lattice, which in turn affects the stability of the amorphous solid dispersion. The enhancement of bioavailability in this approach was driven by both thermodynamic and kinetic forces [68].

Steric hindrance led to the creation of a larger surface area, which in turn inhibits crystallization and prevents the nucleation of crystal growth. The Noyes–Whitney equation was a suitable tool for establishing a correlation between surface area and dissolution, as the two variables exhibit direct proportionality [69]. The first step of dissolution involved the wetting of the molecule, a process that was facilitated by water-soluble polymers. Despite not achieving a complete dissolution release profile, the generation of a supersaturated solution and improved gastrointestinal transit time enhanced the absorption kinetics of the molecule. Additionally, amorphous solid dispersion (ASD) improved the permeation rate by promoting the spontaneous formation of microparticles, nanoparticles, or micelles in the gastrointestinal tract.

Polymers have an important role in ASD formulations and should belong to the “Generally Regarded as Safe” (GRAS) category of food and pharmaceutical ingredients. The FDA’s inactive ingredient database provided lists of excipients/polymers along with their percentage of safety. When selecting an appropriate polymer for ASD, factors such as the physicochemical properties of the drug, the production process, and manufacturability served as the basis. Properties such as molecular weight, polymer type, polydispersity properties, polymer concentration in the formulation, drug solubility, glass transition temperature (*T_g_*) or melting point, hygroscopicity, particle size and distribution, compatibility with active pharmaceutical ingredients and excipients in the formulation, the presence or absence of polymer chemical interactions, mechanical properties, and chemical stability, were also considered [70]. The ratio of the drug to polymer was considered based on properties such as their ability to be processed into tablet or capsule dosage forms while maintaining comfort and convenience. The ASD system played a role in keeping the amorphous drug in the final dosage form. This can be achieved by using low drug strength and high polymer levels. However, unexpected results were avoided by paying attention to the physicochemical interactions such as the thermodynamics of the crystallization or destabilization driving forces, which depend on the loading capacity of the drug, drug–polymer solubility and miscibility, as well as glass transition (*T_g_*) [71]. The advantages of formulating drugs in the ASD system included increasing the rate of dissolution and physical stability. Table 2 summarizes some recent studies of poorly water-soluble drugs employed by the ASD system. There are some methods available for preparing the ASD system as well as the results or the advantages of the ASD system.

### 3.1. Polymers as Carrier Matrix

Polymers consist of repeating units known as monomers that are chemically bonded together, creating an extended structural framework. The integration of amorphous drugs into these cross-linked networks inhibits their molecular mobility due to complicated three-dimensional architectures with multiple interchain or intrachain cross-linkages. As a result, polymers can prevent devitrification by retaining the stability and solubility of the amorphous form throughout the product’s shelf life [92,93,94,95].

#### 3.1.1. Inhibition of Drug Crystallization

Determining the suitability of a drug to form the amorphous phase is crucial before constructing the ASD-based formulation. Glass forming ability (GFA) can provide a qualitative estimate of a medication candidate’s tendency to devitrify. These characteristics could clarify the eligibility for amorphous dosage forms based on physical stability.

GFA and fragility were used as indicators of the prognosis of ASD [96]. The GFA of amorphous drugs, which was defined as the ease with which materials passed through vitrification on cooling, was proposed to have an inverse relationship with crystallization [97]. Several techniques, including reduced *T_g_*, cooling rate dependency, and the crossover point of the heating/cooling rate dependencies of the crystallization temperature, were published in the literature to quantify the GFA of a pharmacological molecule [98].

The “fragility” of a liquid, which was closely related to its GFA and referred to as the “sensitivity” of the structure to temperature change, was used to estimate the kinetic behavior of a supercooled liquid. Strong “liquids” have been identified as being effective glass formers with greater Tm viscosities and resistance to structural alterations. However, the fragile counterparts were weak glass formers, showing lower viscosity at Tm and permitting greater structural alterations with temperature change. By examining the dependence of *T_g_* on the heating rate (q), in differential scanning calorimetry (DSC) studies, the fragility (m) of an amorphous drug was estimated [99,100,101].

Amorphous drug crystallization was a two-step process that occurs simultaneously. The first and second phases were nucleation and crystal growth, which took place at lower and higher temperatures, respectively. A supersaturated medication solution also promoted crystallization. However, it was not the only prerequisite. To overcome the high interfacial tension between microscopic particles, a certain quantity of energy (known as activation energy) was required. As a result, nucleation could not begin until a particular level of supersaturation was reached to supersede the energy barrier. The metastable zone was the range of supersaturated concentrations where no nucleation occurs, and a wise choice of polymeric excipients widened this region by decreasing interfacial energy [102]. Polymers that increase aqueous solubility (by limiting the precipitation of the dissolved medication) reduced the free drug concentration available for nuclei formation, slowing the nucleation rate [103]. The polymer also raises system viscosity, which may change the frequency of atomic or molecular movement at the nucleus’s surface [104]. Furthermore, due to their complex, big, and flexible structures, high molecular weights, as well as the ability to exist in numerous conformations, polymers have sufficiently high configurational entropy. These considerably minimize the possibility of drug recrystallization by lowering the free energy of ASD [105,106].

#### 3.1.2. Antiplasticization

The hardening of a substance or reduction in plasticity was known as antiplasticization [106]. This was described in thermodynamics as a phenomenon that causes an increase in *T_g_* material, thereby increasing the free energy to transform the amorphous drug into crystalline form. The combination of two materials with different *T_g_*s will produce the final *T_g_* of the mixture which falls between their values [107]. When a low-*T_g_* amorphous drug was molecularly combined with a high-*T_g_* polymer, an ASD system with a *T_g_* intermediate of these two components was formed. In other words, the polymer is plasticized, but the drug’s *T_g_* increases due to antiplasticization [108]. According to a study by Sathigari et al. on the stabilization of amorphous efavirenz, the anti-plasticizing effect of the polymer, which raises the system’s viscosity and lowers the drug–molecule diffusion required to form a crystalline lattice, contributed to the drug stable in a solid dispersion with Plasdone S-630 as the carrier. However, the *T_g_* values determined through experiments sometimes differed significantly from those predicted by theory [109]. This was attributed to the suboptimal mixing of the drug and polymer, leading to no volume additivity [110].

#### 3.1.3. Intermolecular Interaction of Drug–Polymer

According to Paudel et al. [111], weak forces such as van der Waals interactions, H-bonds, electrostatic attractions, ionic interactions, and hydrophobic interactions enable the bonding between drug and polymer molecules. These intermolecular bonds restrict the movement of drug molecules within the polymer matrix and contribute to the stability of amorphous solid dispersion systems. Miscibility and the drug–polymer ratio influenced the strength of the intermolecular interactions. The interactions of drug–polymer can be identified using IR and Raman spectroscopy. Therefore, it was concluded that ASD formulations can be influenced by a certain degree of interaction [112].

#### 3.1.4. Molecular Mobility Suppression of Amorphous Drugs in Amorphous Solid Dispersion

The improved physical or chemical stability of amorphous drugs in an ASD system can be described in terms of molecular mobility. Polymer molecules have the ability to limit the molecular mobility of amorphous drugs when used as a carrier. As a result, a mechanistic analysis of the reduced crystallization tendency was required to assess stability. To evaluate molecular mobility in glass systems, solid-state nuclear magnetic resonance (ssNMR), DSC, and dielectric spectroscopy were often utilized. Stronger drug–polymer interactions (ionic or H bonds) decrease the mobility of amorphous drug molecules, potentially delaying crystallization initiation time and reducing the rates [113]. Another intriguing study discovered that the relaxing period of the drug increases with higher polymer content, leading to enhanced stability attributed to the limited molecular mobility of amorphous medications [114].

Various polymers have been used to develop the amorphous drug formulation through the amorphous solid dispersion method. A comprehensive list of polymers commonly used in amorphous solid dispersion is shown in Table 3.

## 4. Preparation of ASD

**Figure 2:**
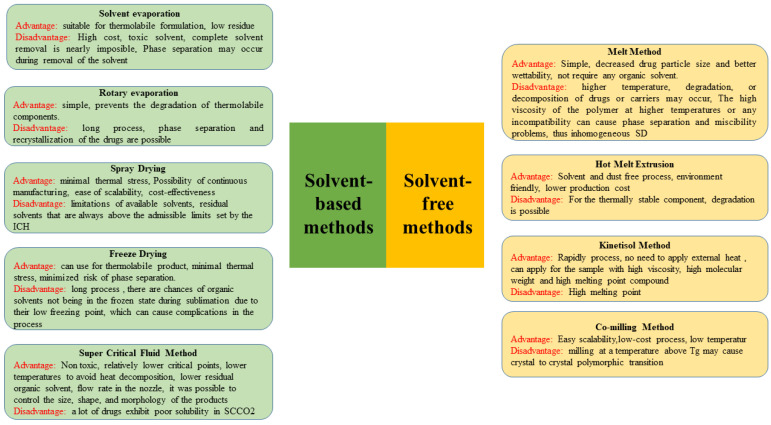
Schematic breakdown of the different methods used to prepare the ASD system [137,138].

Different preparations affected the physicochemical properties of ASD formulation [84,85]. Generally, preparation was divided into two main approaches, namely, solvent-free and solvent-based methods (Figure 2).

### 4.1. Solvent-Based Methods

There are several solvent-based methods for preparing the ASD system, such as spray drying, solvent evaporation, and freeze drying. In the solvent method, removing the solvent is very necessary until the acceptable levels are within the guidelines of the International Conference on Harmonization (ICH) Q3 (R5) [139]. This method is suitable for the polymers applied in the solvent-free or melt method due to their high melting point. However, the solubility of the drug and the carrier should be sufficient as an important prerequisite of this method [140]. In some cases, finding a suitable non-toxic solvent is difficult because the drugs are hydrophobic, while carriers/polymers are hydrophilic [141]. The solvents commonly used in ASD systems are methanol, ethanol, and acetone [142]. The preparation using these methods is challenging because the phase separation occurs during the removal of the solvent. The heating process in the solvent removal can increase molecular mobility, causing phase separation [143]. For human pharmaceutical applications, it is possible to explore the use of less toxic and safe solvents, such as supercritical/near-critical CO_2_, as alternatives in the preparation of ASD. These solvents offer potential advantages in terms of minimizing toxicity and ensuring safety [144,145,146]. Despite its limitations, the solvent method remains valuable at laboratory levels due to its ability to address the primary challenges associated with the melting method, particularly the potential decomposition of drugs and polymers at high temperatures [147].

#### 4.1.1. Solvent Method

Solid dispersions are acquired through the evaporation of the solvent from a solution comprising the drug and carrier, and employing the solvent method. In the development of ASD, a critical factor to consider is the selection of an appropriate solvent system. The primary challenge associated with this method lies in obtaining a solvent system capable of effectively solubilizing the drug–polymer system while maintaining compatibility with the formulation [111]. A notable advantage is its ability to prevent the drug and polymer from undergoing decomposition due to heat, making it suitable for thermolabile formulations and low-melting-point drug substances. This advantage is similar to the freeze-drying method, which does not rely on heat for the process [148]. Using the same solvent for both the drug and polymer presents a challenge when there are notable differences in polarities. An excessive amount of polymer can also lead to an imbalanced ratio, resulting in a reduced drug loading capacity. Consequently, increased polymers can negatively impact the body’s tolerance, underscoring the importance of maintaining an appropriate balance between the two components [25]. The use of excessive solvents causes the process to be more expensive. An additional challenge arises from the potential occurrence of phase separation during the process of solvent removal. Achieving complete removal is exceedingly difficult, necessitating an increase in evaporation temperature. However, the utilization of high temperatures accelerates phase separation due to the enhanced mobility of drug molecules and polymers [149]. The solvent used in this method must be non-toxic and have sufficient solubility for the drug and carrier. Meanwhile, surfactants such as Tween 80, SLS, Poloxamer, PVP K30, and PEG 6000 can be added to increase drug solubility [148,150].

#### 4.1.2. Rotary Evaporation

This method is carried out by evaporating organic solvents at moderate temperatures using a rotary evaporator to prevent degradation of the heat-labile acceptance script components of the solid dispersion formulation. It is used in the manufacture of ASD systems on a small scale [151]. After the solvent is evaporated, the remaining dry sample is subjected to grinding, sieving, and subsequent placement in a vacuum desiccator to eliminate any residual solvent. To address the solubility challenges, mixed solvents are commonly employed. However, this method is comparatively less efficient than alternative solvents due to its prolonged duration, potentially resulting in phase separation and drug recrystallization [148].

#### 4.1.3. Spray Drying

This method is commonly used for preparing ASD systems on a large scale. It can be used to produce particles through a hot gaseous drying agent to convert liquid substances into dry particles [152]. Spray drying employs a nozzle to spray drugs and polymers, necessitating careful consideration of the nozzle type. Commonly utilized nozzle types include the external mixing two-fluid, internal mixing two-fluid, pressure-swirl, and pressurized nozzles. Furthermore, it has been demonstrated that the composition of the solvent during the process significantly affects ASD, even when the drug and polymer are fully dissolved in the selected solvent system [153]. A study conducted by Li et al. found that the addition of water to the solvent system can cause phase separation and drug recrystallization [154,155]. The basis of the process is to remove moisture while exposing the feed product to a hot environment. There are three main phases in this procedure, namely, atomization, the conversion of droplets to particles, and the collection of particles [152]. By utilizing this method, it becomes possible to prevent the separation of drug and carrier phases. Furthermore, it offers significant potential for sustainable manufacturing, featuring simplicity in scalability, excellent molecular dispersion uniformity, cost-effectiveness in large-scale production, and high recoveries [156].

#### 4.1.4. Freeze Drying

The proposed technique can be applied to thermolabile products that exhibit stability in dry conditions but are prone to instability when exposed to water. However, these products demonstrate good stability when stored in a dry state for extended periods. The process of freeze drying, also known as lyophilization, involves combining active ingredients with a carrier dissolved in a solvent. Lyophilization achieves molecular dispersion and facilitates the formation of amorphous systems through the combination of freezing and sublimation. In the case of poorly water-soluble substances, ASD is typically utilized, while freeze drying is commonly performed from an aqueous solution [157,158]. This method has the advantage of limiting temperature stress during the production of solid dispersions of the active ingredients as well as minimizing the possibility of aqueous phase separation immediately after vitrification. Relevant organic solvents for freeze drying are few because the majority have extremely low melting points [159]. This approach also offers the advantage of minimal thermal stress and reduced potential for phase separation. It is important to note that the process is time-consuming, and complications can arise when organic solvents fail to enter the frozen state during sublimation due to their low freezing points [68,160,161].

#### 4.1.5. Supercritical Fluid Method

ASD can be prepared using supercritical fluid technology (SCF), which has several special benefits, such as moderate preparation conditions, environmental friendliness, adjustable processing settings, and high reproducibility [162,163]. Furthermore, more than 98% of all SCF applications use supercritical carbon dioxide (SC-CO_2_) in supercritical process fluids with properties that are non-toxic or inert, inexpensive, and inflammable; have a relatively lower critical point, lower temperatures to avoid heat decomposition, and lower residual organic solvents and flow rate in the nozzle; and the possibility of controlling the size, shape, and morphology of the product [164]. Even though a lot of drugs exhibit poor solubility in SCCO_2_, cosolvents such as methanol can be added to CO_2_ to improve drug solubility. In this method, SCF CO_2_ is primarily employed, inducing expeditious sedimentation of the solid amalgamation, thereby precluding sufficient duration for the segregation of the drug and polymer in the fabrication of solid dispersions [165]. This method forms very stable small particles with a higher surface area for good flow. The solvent evaporation process can also be controlled by adjusting the temperature and pressure conditions to obtain a low residual organic solvent, and the low viscosity of SCF results in high diffusivity and fast solvent evaporation. Disadvantages of using SCF-based methods for ternary system preparation include difficulty in removing residual organic solvents and high capital investment [166].

### 4.2. Solvent-Free Methods

The solvent-free methods are less time-consuming and can achieve a high entrapment efficiency compared to solvent-based methods. Moreover, the amount of drug used in the ASD system can easily be predicted based on the ratio of each component. This method is an environmentally friendly technique and checking the residual solvent in drug products is not required. The method does not use organic solvent to prepare the ASD system and can be located in the stream of “zero waste” chemistry.

#### 4.2.1. Melting Method

The melting method is commonly used to produce solid dispersions by heating the material until it melts, then this is followed by cooling. All materials melt together at a temperature higher than the eutectic point. This eutectic combination has sufficiently high molecular mobility to allow the drug particles to occupy the matrix, resulting in a smaller drug particle size and better wettability. Furthermore, the degree of cooling during this process influences how the drug is incorporated into the matrix [167]. This technique incorporates thermal energy, rendering it unsuitable for the production of heat-sensitive pharmaceuticals. Drugs possessing elevated molecular weights and diverse atom and functional group compositions tend to exhibit increased proclivities for thermal degradation [147]. This homogeneous mixture is first melted and then solidified using various methods, such as freezing, ice bathing, spreading a thin layer over stainless steel, spreading on a plate placed over dry ice, soaking in liquid nitrogen, cryo-grinding, and pouring into a petri dish at room temperature in a desiccator [147]. This approach circumvents the need for solvents, rendering it a more cost-effective and dust-free alternative [168]. The cooling rate employed in this technique plays a pivotal role as it directly impacts the drug incorporation process within the matrix [169]. The application of high heat induces degradation, while elevated viscosity levels lead to the separation of components and hinder proper mixing, thereby resulting in heterogeneous solid dispersions [68].

#### 4.2.2. Hot Melt Extrusion (HME)

Hot melt extrusion (HME) is a modern version of the melting method often used in the manufacture of ASD systems [170]. In the extrusion process, the drug and polymers are mixed into a physical mixture and then extruded under specified conditions. Processing parameters, such as shear force, feed rate, die geometry, barrel design, temperature, and screw speed play an important role in the final product quality [171].

The extrusion process can be carried out at temperatures below the glass transition or *T_g_* of the mixture. However, when the drug is stable in heat, the use of temperatures above the melting point is preferred for the best process and appropriate rheology [172]. The material used in this method must be heat stable to prevent degradation. The technique offers several advantages such as (1) being a solvent-free method; (2) having fewer processing steps due to no material compression and no need to dry the product, making the technique simple, sustainable, and efficient; and (3) that thorough mixing at high shear rates and temperature causes the particles to disaggregate and creates a uniform distribution of fine drug particles in the polymer matrix and molecular level dispersion [173]. This technique offers the possibility of continuous manufacturing, which makes it suitable for large-scale production.

#### 4.2.3. Kinetisol Method

Kinetisols represent an innovative method for preparing contemporary fusion-based solid dispersions. This technique entails using rapidly rotating, constant-volume closed chamber blades that generate substantial friction and shear forces, ultimately producing heat to facilitate the melting of the drug–polymer mixture. Consequently, this approach eliminates the necessity for additional external heating [174]. Rapid heating causes a decrease in the exposure of the material to thermal exposure to avoid degradation [175,176].

Kinetisol finds application in materials characterized by melting points exceeding 200 °C, rendering them unsuitable for employment in the HME method. It proves advantageous for compounds that present challenges in dissolving within organic solvents, thereby circumventing issues encountered with the solvent method. Furthermore, Kinetisol proves highly effective in handling materials exhibiting high viscosity, which commonly gives rise to droplet formation complications in the spray drying method [177]. The operational design of this method caters to batch mode operation at the laboratory scale. However, it possesses the flexibility to be run semi-continuously at an industrial scale, enabling outputs of up to 1000 kg/hr [178,179].

#### 4.2.4. Co-Milling Method

Particle size reduction is known to reduce crystallinity and achieve amorphization. The solubility of pharmaceutical dosage forms, flow characteristics, and content uniformity are improved by reducing the particle size and formation of nanoparticles. Furthermore, energy-intensive co-milling techniques can produce well-mixed drug excipient mixtures with potentially varying degrees of crystallinity. This technique also offers the advantages of easy scalability and a low cost of processing [169]. Milling at lower temperatures improves amorphous tendencies while grinding at temperatures above *T_g_* induces polymorphic crystal-to-crystalline transitions [180]. Hydrophilic excipients also interact with the drug to increase wettability, which increases drug solubility and bioavailability [181]. This method using drug milling technology (ball, hammer, micronized, or nano mill) and drug co-milling with workable conformers is very promising [182]. However, this method is not popular in the pharmaceutical industry [183] due to the frequent risk of residual crystallinity, acting as a seed and inducing crystallization during shelf life [184].

## 5. Characterization of ASD

In-depth analysis of these formulations was necessary due to the characteristics of ASDs and the potential risk of recrystallization. Given the complexity involved, a diverse range of complementary techniques is often required. This is because no single characterization methodology can provide all the necessary information.

### 5.1. X-ray Powder Diffraction (XRPD)

An essential tool for characterizing amorphous solid dispersions was powder X-ray diffraction [185]. This method was used to confirm the presence of the drug in its amorphous state within the solid dispersion [186,187,188]. Recent developments in XRPD instrumentation and software have significantly contributed to a better understanding of the molecular behavior of amorphous drugs in amorphous solid dispersion under stressed conditions. For example, XRPD equipped with variable temperature (VT) or humidity control has proven valuable in providing information under non-ambient conditions [189]. Zhu et al. employed in situ small-angle/wide-angle X-ray scattering to investigate the crystallization kinetics of a naproxen ASD system at various temperatures [190]. Additionally, there has been an increased recognition of the significance of utilizing the atomic pairwise distribution function to measure the degree of amorphization caused by crystalline drugs [191]. Nollenberger et al. demonstrated the impact of minor modifications to polymer structure at the molecular level on the release characteristics of the finished product using pairwise distribution function analysis [192].

### 5.2. Thermal Analysis

DSC and thermogravimetric analyses were the two most frequently employed thermal analyses (TAs). However, dynamic mechanical analysis and isothermal microcalorimetry were utilized in the pharmaceutical sector for regular examination. These cutting-edge TA techniques, including DSC, offer valuable insight into various molecular processes that take place in the solid dispersion, such as glass transition, polymorphic transition, crystallization, structural relaxation, molecular mobility, as well as miscibility between drug and polymer [193]. *T_g_* and heat capacity analyses were applied by Mahajan et al. to measure the amount of amorphous material present in carvedilol tablets [194]. Additionally, the increased sensitivity of fast-scan DSC allows for the separation of overlapping thermal events, providing an additional advantage [195]. Differential mechanical thermal analysis offers a means to explore the relaxation transitions, the viscoelastic properties of polymers, and miscibility in binary or ternary systems [196].

The development of increasingly advanced sensors in recent years has made real-time solid-state characterization, as a function of temperature change, achievable. With the use of methods such as VT-XRPD, VT molecular spectroscopy, and VT-ssNMR, amorphous drugs’ molecular orientation, structural relaxation in ASD systems, and their interaction with polymers were examined in greater depth. In particular, the localized TA technique known as nano-TA, when combined with atomic force microscopy, can produce high-resolution images of the thermal behavior of amorphous drugs. Incorporating a tiny heater with a topographic resolution of around 5 nm onto a microfabricated silicon-based probe enabled the measurement of thermal characteristics at a nanometer scale [197].

### 5.3. Spectroscopy

Among the vibrational spectroscopic techniques, Fourier transformed IR spectroscopy and Raman spectroscopy, when combined with attenuated total reflectance and/or diffuse reflectance, have proven to be the most effective [198,199]. These approaches have been applied in the pharmaceutical industry for a variety of purposes, including phase transition, polymorph identification, recrystallization stability, evaluation of various manufacturing processes for solid dispersions, phase separation, and the type and degree of drug–polymer interaction [115,200]. By investigating band vibrations, these methods provided details on structural and molecular conformation in the solid state. Furthermore, the interior structure of molecules and crystals can be identified using the potent light-scattering technique known as Raman spectroscopy. Studying the low-energy lattice vibrations connected to various crystal packing configurations provided information into the crystal packing [201]. Raman spectroscopy has been employed by Furuyama et al., as a mapping tool to differentiate between troglitazone’s crystalline and amorphous forms in solid dispersions [202]. Sinclair et al., utilized FT Raman spectroscopy to study the recrystallization kinetics of an amorphous solid dispersion of ibipinabant [203]. Additionally, it was applied for the identification of trace crystallinity that could have been missed by XRPD or high-sensitivity DSC [204].

### 5.4. Water Vapor Sorption

Water vapor sorption has frequently been used to investigate the behavior of crystalline and amorphous materials when exposed to moisture. To evaluate the moisture sorption data and gain understanding into drug polymer-water interactions, ternary FH interaction theory was employed [205]. Furthermore, when combined with other methods such as DSC, Fourier transform IR spectroscopy, and nuclear magnetic resonance (NMR), it provided a variety of data on molecular level attributes such as degree of amorphization, surface properties, phase transitions, critical relative humidity for glass transition and crystallization, as well as physical stability of materials [206,207,208]. The combination of near IR spectroscopy with dynamic vapor sorption allows for an understanding of the desorption behavior of amorphous drugs before and during its crystallization, as a function of temperature and relative humidity [209].

### 5.5. Solid-State Nuclear Magnetic Resonance

ssNMR is a nondestructive method that provides information about amorphous solid dispersions in both qualitative and quantitative forms, and it offers comprehensive one- and two-dimensional structural data based on NMR relaxometry, spectroscopy, and imaging [210]. The size of the drug polymer domain in solid dispersions was predicted by correlating the relaxation period with the length scale of the spin diffusion. For instance, the spin–lattice relaxation time, T1, had values in the range of 1 to 5 s, which equated to a domain size of about 20–50 nm. T1r (spin–spin relaxation time) values between 5 and 50 ms suggested a length scale of approximately 2–5 nm. These relaxation time measurements allowed for accurate forecasts. A single value of 1 H T1 and T1r in amorphous solid dispersions indicates a domain smaller than 2–5 nm. A size of about 5–20 nm was exhibited by several T1r values but the same T1 value. For drugs and polymers, domain sizes greater than 20–50 nm result in distinct T1 and T1r values. Compared to DSC which only provided single *T_g_* values for domain sizes smaller than 20–30 nm, this approach is substantially more sensitive. To improve the stability of amorphous solid dispersions and prevent phase separation during the product’s shelf life, ssNMR relaxometry better comprehended drug–polymer intimacy in the solid dispersion and preventing phase separation [211]. ^1^H transverse magnetization relaxation T2 measurements provided information regarding the phase composition and mobility of polymer molecules in solid dispersions [212]. Meanwhile, ^13^C cross-polarization magic angle spinning NMR experiments were used when no differences were observed in the XRPD pattern [213]. Additionally, NMR tests were utilized to investigate the recrystallization of amorphous troglitazone in solid dispersions made using various techniques. A useful complement to analytical techniques for analyzing the kinetics of polymer mobilization and water penetration was the NMR microimaging approach [214].

### 5.6. Inverse Gas Chromatography

A developing method, inverse gas chromatography, has been utilized to examine the surface characteristics of amorphous solid dispersions [215]. This technique was employed to investigate molecular mobility, amorphous transition or recrystallization, and molecular relaxation. Furthermore, it is particularly needed for the identification of batch-to-batch variation in amorphous solid dispersions using the same or different techniques [216]. A study of the increased molecular mobility on a material’s surface compared to its bulk provided insight on the interactions between moisture and the recrystallization of amorphous drugs [217]. Inverse gas chromatography was employed by Hasegawa et al. to explore structural relaxation at the surface of solid dispersions [218]. Furthermore, it was discovered that structural relaxation occurs more quickly at the surface than in the bulk due to increased molecular mobility. The kinetics of crystallization on the surface of solid dispersions were used to predict the physical stability of amorphous products [219].

## 6. Dissolution of ASDs

**Figure 3:**
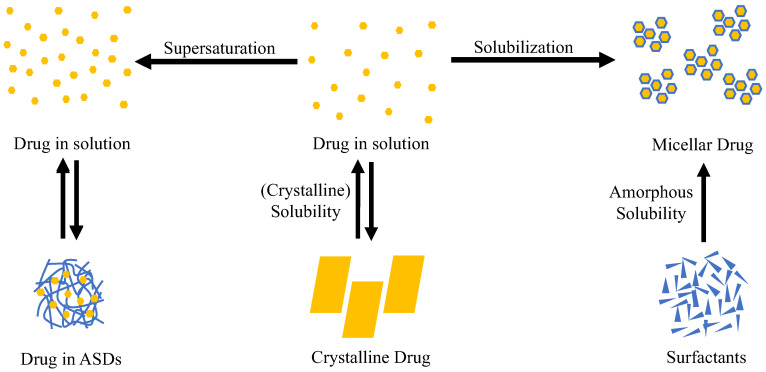
Classification of the physicochemical concepts of solubility, supersaturation, and solubilization. There are two types of API equilibria in solution: (1) the equilibrium between crystalline drugs and drugs in the solution, known as crystalline solubility, and (2) the equilibrium between drugs in amorphous liquid phase separation (ALPS) and drugs in solution. Equilibrium 2 has a larger concentration of molecularly dissolved drugs (referred to as supersaturation) than equilibrium 1. Solubilization, for example, by surfactants, does not result in an increase in the concentration of molecularly dissolved drugs. Adapted from data presented originally in Ref. [220].

Dissolution was the first crucial stage in the bioavailability cascade. In this review, a separate chapter was devoted to the transformation of ASDs from solid to liquid, despite the inherent interconnectedness between this phase and subsequent stages leading to the absorption of active pharmaceutical ingredients. The dissolution of drugs is always attributed to solubility. In current literature, the terminology of solubility, supersaturation, and solubilization of API in solutions was different as shown in Figure 3. Solubility of API generally refers to molecularly dissolved molecules of an API in an aqueous solution. Supersaturation is the effect of the dissolution of more API than its crystalline equilibrium solubility. Meanwhile, solubilization is the increase in solubility of API with surface-active agents such as surfactants [220].

Studies suggested that ASDs could exhibit sparse or incomplete disintegration. This phenomenon was demonstrated in a study involving phenytoin and probucol as model medicines, along with various polymers [221]. The absence of dissolution and the creation of colloidal states was discovered depending on the polymer and drug load. Furthermore, Aleandri et al. [222] highlighted that the second dissolution stage, where the drug was liberated from colloidal states into the molecularly dissolved form, should be successful enough to facilitate absorptive flux across the intestinal epithelium.

### Dissolution Mechanism

According to the ideas of Simonelli et al. [223], Craig [224] created the notions of carrier and drug-controlled release in ASD. In the case where the polymer does not dissolve in the media and instead forms a highly viscous gel layer, the carrier becomes a limiting step for drug release. This results in the slow diffusion of the API molecule compared to pure solvent. Meanwhile, when the polymer dissolves into the dissolution media without the formation of a gel layer and the drug particles are exposed to the medium, the dissolving process is drug-controlled. Furthermore, it is possible for both of these rate-controlling processes to occur simultaneously [68]. Despite the drug release concept requiring heterogeneous dissemination in the carrier matrix, this strategy could be regarded suitable even for homogeneous dispersions. When polymer dissolves more quickly than the drug, crystallization can occur in its absence. Conversely, the formation of a viscous gel layer facilitated the dissolution of crystalline drugs [225,226].

Sun and Lee [227] proposed a different dissolution theory that distinguished between soluble and insoluble carriers as model pharmaceutical molecules. Based on the solubility of the carrier in the release medium, comparisons were made between diffusion-controlled (insoluble in carriers) and dissolution-controlled (soluble in carriers) releases. In the first instance, there was a quick transition into dissolved or colloidal phases. This rapid liberation and subsequent dissolution of the amorphous API can result in supersaturation. The presence of the dissolved polymers prevents the supersaturated solution from rapidly crystallizing [228]. Meanwhile, in a dissolution-controlled release, the API Is continuously diffused from the matrix of polymers into the release medium, resulting in a carrier-controlled release mechanism. This mechanism was driven by the gradient in drug concentration between the release medium and the carrier. As a result, the API concentration in the release medium will not exceed that of ASD. When the concentration in the release media was reduced, more drug diffused from the carrier into the medium. In other words, the carrier functions as a depot, controlling the maximum drug concentration in the release medium. This concept aligns with a study by Han and Lee [162] that crystallization was not induced when drug concentrations in the dissolving medium fall below a critical point. These results are consistent with Baghel et al.’s review on polymeric ASD formulations, where ASD dissolution was divided into two scenarios, namely, rapid dissolution followed by crystallization from the solution of increased apparent solubility [25].

In the dissolution-controlled scenario, there was no report by Sun and Lee on the creation of a drug-rich phase. However, Saboo et al. [229] stated that colloidal states formation was possible. A congruent release of polymer and API (dissolution-controlled release) was proposed to be essential for particle formation by ALPS, and this was consistent with the concept of dissolution-controlled release.

The study by Indulkar et al. [230] established that carrier-controlled release can result in the creation of drug-rich particles. Furthermore, isotope scrambling in conjunction with NMR spectroscopy was employed to investigate the genesis of drug-rich particles using nifedipine with HPMC or PVPVA as examples. Two theoretical principles for the formation of drug-rich particles were distinguished into (1) the carrier-controlled molecular dissolution of the drugs and subsequent phase separation when the concentration exceeds the amorphous solubility, and (2) the dispersion of drug-rich domains present in the solid state of ASD. The first applicability of the mechanism for the ASD under investigation was demonstrated experimentally.

Puncochova et al. [225] developed a dissolving mechanism based on imaging investigations. On the basis of three polymers and aprepitant as a medicinal ingredient, ASD-dissolving mechanisms were studied using attenuated total reflection Fourier transform infrared (ATR-FTIR) and NMR imaging. The polymer matrix release process discovered was water entering the tablet, swelling of the polymer, diffusion of the medicine from the swelling matrix, and disintegration of the polymer. It was observed that the gel layer can keep the API supersaturated. As a result, rapid polymer degradation can result in drug crystallization. In this instance, API dissolution was regulated by diffusion through the gel layer (carrier-controlled dissolution) and, in the early stages, by water ingress (when no disintegrant was utilized). This followed the study of Dahlberg et al. [214] where it was shown that the rate of water ingress has no direct effect on release kinetics. Instead, drug release correlates with polymer mobilization kinetics.

**Figure 4:**
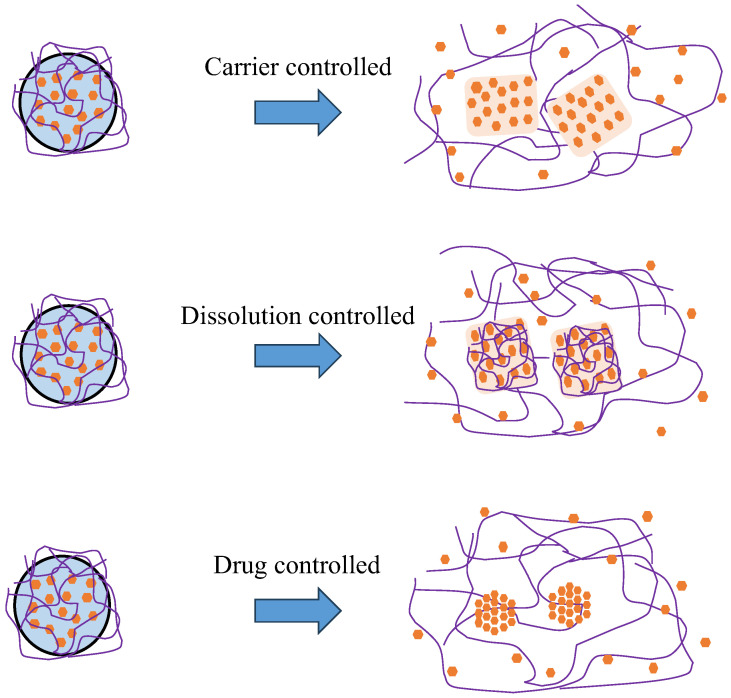
The mechanism of ASDs after being dispersed into dissolution medium adapted from data presented originally in Ref. [220]: (1) In the event of a carrier-controlled release, drug molecules will diffuse through the polymer, maybe via a highly viscous gel layer on the surface of ASD particles. ALPS occurs when dissolved drug concentrations exceed the amorphous solubility, resulting in the production of drug-rich particles. (2) In the event of controlled release, the drug and polymer dissolve congruently, resulting in rapid dissolution and the creation of drug-rich particles. The polymer may help to keep the supersaturated solution stable. (3) In the case of drug-controlled release, the polymer dissolves out of the ASD and the remaining drug regulates the pace of dissolution. Supersaturation will not occur if the residual drug is not stable in the amorphous state without polymer, i.e., crystallizes.

In conclusion, there was growing evidence from the literature of primarily three mechanisms by which ASDs dissolve, as shown in Figure 4.

(a) *Carrier-controlled release*. Water penetrates the polymer, causing the creation of an extremely viscous gel layer through which the drug molecules should diffuse. The concentration in the dissolving medium was controlled by that of the drug in the ASD and the volume of the release medium, resulting in a delayed release. Furthermore, drug-rich particles were formed when the amorphous solubility limit was exceeded.
(b) *Congruent release* (*dissolution-controlled release*). Drug and polymer were released quickly and simultaneously into the dissolving solvent, creating a noticeable supersaturation effect. In this case, the polymer played a crucial role in stabilizing the supersaturated condition in solution. The overall medication dose and the volume of the release medium regulate the supersaturation concentration.
(c) *Controlled drug release.* The polymer dissolved in the medium, leaving the amorphous drug to dissolve at a drug-controlled rate. There is a possibility of the crystallization of the drug when employing this technique. Furthermore, the formation of drug-rich particles was theoretically possible when the amorphous state of the medications was stable enough. However, no experimental data pertaining to this review were identified.

## 7. Stability of Amorphous Solid Dispersion

In contrast to crystalline solids, which are structured in a three-dimensional array, their amorphous counterparts had short-order arrangements of molecules. When compared to their crystal form, amorphous materials had perfect pharmacological characteristics, including greater solubility and higher kinetic solubility. Furthermore, a well-developed system existed in a supersaturated state in vivo, leading to enhanced drug exposure. The primary weakness of ASD, despite all of its advantages, was a lack of physical and chemical stability, which frequently creates obstacles for the creation and commercialization of the product [159,231]. The primary causes of this instability were (1) a lack of potential technologies to predict the stability of formulation; (2) a lack of knowledge about the physicochemical properties of API, additives, and polymers; and (3) a lack of knowledge on manufacturing technology setup. These elements should be considered to mitigate the risks associated with physical instability.

There are numerous approaches to discuss the mechanistic understandings and instability of ASD, which was created using a variety of strategies. The conventional method for forecasting stability was to anticipate it under stressful circumstances in accordance with ICH principles. Long-term stability typically provides a good view of the solid-state properties, as well as the physical and chemical integrity, of an ASD formulation [232]. According to ICH Q1 recommendations, a typical set of stress settings typically comprised of 2–8 °C chilled, 25–60% RH, 30–65% RH, 30–75% RH, and 40–75% RH with time points ranging from 1 day to 6 months or 2 years. The sample was examined at every time point and under each stress state using XRPD, DSC, and/or FTIR, as shown above. During the late stages of drug development, years of long-term stability testing are conducted to determine the shelf life of the product using either PXRD or DSC, focusing on actual stability measurements rather than predictions [233]. In addition to stability assessment, maintaining supersaturation during in vivo dissolution testing is crucial for the ASD formulation to improve solubility and maximize drug absorption in order to develop effective pharmacological products. Suspension stability was also important in preclinical animal experiments. The ASD stability was conducted to support toxicological and/or pharmacokinetic studies, and the ideal suspension vehicle should enable the API to remain amorphous for 4–6 h at ambient temperature [234,235].

### 7.1. Factor Affecting Stability

In the meta-stable state of ASD, there was a possibility of spontaneous conversion back into a more stable crystalline form. This conversion was influenced by the interaction of the drug with a polymer, which reduced molecular mobility and molecular coupling. Processing and storage conditions, including variables such as temperature and relative humidity (RH), played a significant effect on thermodynamic (responsible for nucleation and crystal development) and kinetic (molecular mobility) aspects [236,237].

#### 7.1.1. Thermodynamic Aspect

When ASD was modeled as a glassy solution of a poorly soluble drug in a hydrophilic polymer with a high glass transition, the solid-state alterations became visible at the molecular level. The rate of temperature decreased, and kinetic aspects were inversely proportional to the thermodynamic driving force, which was responsible for crystallization. This indicates that as the rate of supercooling increases, the thermodynamic force intensifies, leading to an increased kinetic barrier to crystallization and decreasing molecular mobility [238]. After the formation of a stable nucleus due to the thermodynamic driving force of nucleation, a bulk of crystalline material was obtained through crystal growth [239].

#### 7.1.2. Molecular Mobility

Kinetic stability of the product significantly reduced the possibility of drugs’ re-crystallization in the ASD system. The stabilization, which held the transitional and rotational movements of the molecule and permitted the API molecule to diffuse surface integration, was greatly influenced by molecular mobility [240,241].

#### 7.1.3. Temperature

The stability of ASD was influenced by temperature, since crystallization is a temperature-dependent process. When the glass phase transitioned to the liquid phase at a temperature above *T_g_*, the rapid phase separation and crystallization of ASD occurred [242]. The *T_g_* 50 °C rule advised that ASDs at least 50 °C below their *T_g_* should be stored. However, it does when crystallization is triggered by relaxation. An alternative option for storing the ASD is the Kauzmann temperature (Tk) [243,244]. At this temperature, molecular mobility can be completely stopped.

#### 7.1.4. Moisture

The interactions between the wet and API or the polymer have a significant impact on the ASD stability. Amorphous forms absorb more water than their crystalline counterparts because they have a higher kinetic solubility [245]. The presence of water in ASDs exhibits a plasticizing effect, reducing the transition temperature and accelerating crystallization. Plasticizers had impacts on the system’s numerous properties by reducing strength, lowering the temperature at which viscosity transitions occur, and increasing molecular mobility, thereby leading to a rise in physical and chemical instability [246]. The polymers also caused the dispersed API to become less mobile when they came in contact with water or moisture. This is because they formed a hydrogen bond with the water, polymer, and API. Techniques such as differential scanning calorimetry (DSC), near-infrared methods, and Fourier Transform (FT) can be employed to detect and analyze these interactions (FT) [247].

## 8. ASD for Cancer Therapy

Chen et al. [248] extracted five major compounds from the total 23 ioflavonoids derived from *Selaginella doederleinii* in amorphous solid dispersion form using the solvent evaporation method. *The compounds included amentoflavone, 2″,3″-dihydro-3′,3‴-biapigenin, robusta flavone, 3′,3‴-binaringenin, and delica flavone.* The polymers used for the formulation were poloxamer 188, PVP K30, EPG 4000, and PEG 6000. However, only PVP K30 produced a homogeneous solid dispersion (Table 4). This is because the mixture with poloxamer 188, PEG 4000, and PEG 6000 formed a sticky mass solid dispersion.

Pharmacologically, TBESD-ASD has anti-tumor properties, and the activity was detected by conducting in vivo tests on mice with A549 cell xenograft using doses of pure TDS and TDS-ASD at 200 mg/kg. This was observed from TGI (Tumor Growth Inhibition) which increased from 29.48% to 58.44% after making TBESD in the form of amorphous solid dispersion. TBESD-ASD also decreased the number of MVD tumor xenografts by 52.20% compared to a reduction of 24.30% with pure TBESD. Based on the MVD value, it was concluded that TBESD-ASD exhibits higher anti-tumor and anti-angiogenesis effects compared to pure TBESD.

Despite paclitaxel belonging to BCS class 4, it has also been used to treat cancer. Moes et al. developed an oral dosage form of paclitaxel as an amorphous solid dispersion using the freeze-drying process and PVP K30 as a carrier. The formulation boosts the dissolving rate, provides superior solubility, and prevents crystallization. Polymer PVP primarily inhibited crystallization and promoted solubility, while SLS improved wettability, thereby accelerating dissolution. The amorphous solid dispersion of paclitaxel, known as ModraPac001 10 mg, was formulated as a capsule for clinical studies and exhibited (nearly) the same pharmacokinetic characteristics as the premix solution of low-dosage paclitaxel, which was already utilized for anticancer therapy.

Choi et al. [249] developed a solid dispersion system for PTX using the solvent evaporation method. Based on the solubility and dissolution tests, the best SD was obtained on the SD4 (consisting of PTX, polyvinylpyrrolidone/vinyl acetate (PVP/VA), D-α-tocopheryl polyethylene glycol 1000 succinate (TPGS), Aerosil^®^ 200) and SD9 formulations (consisting of PTX, TPGS, and Aerosil^®^ 200). Anticancer activity was assessed using MTT test on breast cancer (BT-474, MCF-7 and SK-BR-3) and non-cancer cells (RAW 264.7) at drug concentrations of 0.1, 1, 5, 10, and 20 µg/mL. The results showed that the concentrations of both SD formulations were non-toxic to normal cells. The cytotoxicity of SD4 and SD9 on BT-474, MCF-7, and SK-BR-3 cells were 3.0 and 2.8, 4.0 and 4.1, as well as 5.6 and 5.0 times higher than PTX alone. These results indicated that SD4 and SD9 had greater cytotoxicity in breast cancer cells compared to PTX alone.

Liu et al. [250] developed an oral formulation of RA-XII in order to create a therapeutically effective anticancer drug. However, due to poor solubility and limited permeability, RA-XII demonstrated minimal oral bioavailability in mice. For effective distribution of this drug by oral administration, Liu et al. employed a natural deep eutectic solvent (NADES) in the investigation. The technique employed was amorphous solid dispersion utilizing PVP polymer. This formulation enhanced cytotoxicity in vitro, dissolution rate, perceived solubility, and homogeneity. When compared to pure RA-XII in 0.5% CMC-Na, the oral bioavailability of this drug in NADES and ASD solutions was increased by approximately 11.58 and 7.56 times, respectively. According to pharmacokinetic studies conducted in vivo, the ASD of RA-XII inhibited cell proliferation of all RKOs more effectively than free RA-XII. The therapeutic effect of RA-XII was more pronounced in cases with severe basal cancer when formulated as an ASD.

Kumar et al. [251] conducted a study on the formulation of anticancer IIIM-290 in solid dispersions using PVP K-30, xanthan gum, and PEG-PPG-PEG polymers, and the method employed was solvent evaporation. The aim was to improve solubility, dissolution, and oral pharmacokinetics. SD IIM-290 increased the solubility by 17-fold compared to pure IIM-290. This has an effect on the enhancement of anticancer activity. Based on an in vivo study conducted on rats with Ehrlich ascites carcinoma, the samples were divided into four groups. Group I was given IIM-290 at a dose of 50 mg/kg, and groups II, III, and IV were administered SD IIM-290 at doses of 20, 50, and 75 mg/kg, respectively. The results showed that SD IIM-290 increased plasma exposure by 1.9-fold and exhibited a tumor inhibition of 31–43% compared to pure IIM-290 with a rate of 24.75%.

## 9. Discussion

**Figure 5:**
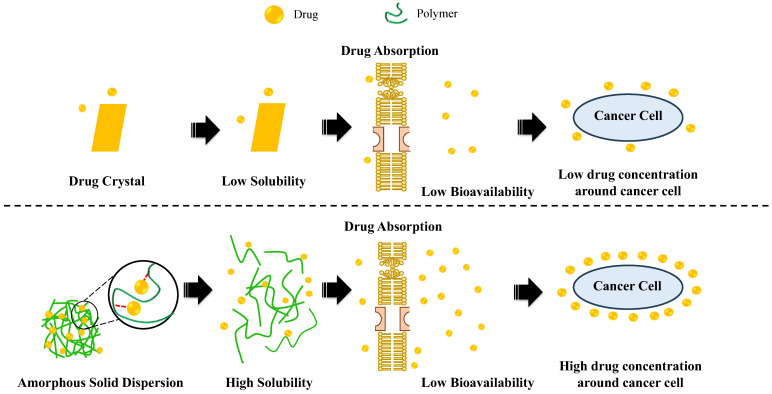
The speculated mechanism of bioavailability improvement from amorphous anticancer drugs in amorphous solid dispersion. (1) Anticancer drugs in crystalline form have low solubility leading to low bioavailability. (2) Anticancer drugs in amorphous solid dispersions have high solubility. This increase in the concentration of molecularly dissolved drugs increases absorption; therefore, they have high bioavailability.

A crucial challenge in the uptake of dissolved ASD was the absorption of anticancer drug particles that were molecularly dispersed. Previous study reported that the absorption of poorly water-soluble drugs, including anticancer medications, was enhanced only when API was in a supersaturated state [252]. Meanwhile, the presence of solubilized API, such as micelles from endogenous bile salts or surfactants in the formulation, impeded the transport of anticancer drugs [253]. The mechanism of drug absorption commonly occurred by passive diffusion. Based on the Fick law of diffusion, the drugs will move due to the concentration gradient from a higher concentration to a lower concentration of drugs until equilibrium is reached. In the case of drug absorption, the soluble drug in the aqueous compartment of the body, such as interstitial space, will move through aqueous pores in the endothelium of blood vessels. Thus, the solubility of the drug in the aqueous compartment of the body significantly affects the amount of drug absorption via passive diffusion. The drug absorption of anticancer drugs crystal could be lower compared to that amorphous state due to their poor water solubility. Meanwhile, in the ASD of anticancer drugs, the amount of drug absorption would be higher because the solubility of drugs was improved and the supersaturated solution in the aqueous compartment of the body was maintained by the intermolecular interaction of drug and polymer [254]. According to studies, the transport mechanisms are passive diffusion, which had been demonstrated to improve in vivo bioavailability [255]. Shi et al. [256] reported that the concentration of molecularly soluble drugs in the release medium was not significantly affected by the increase in solubility of the drug by micellization. This suggested that only higher concentrations of molecularly dissolved drugs (true supersaturation) were significant for increased permeation rates [257,258,259]. As a result, it was assumed that the absorption of poorly water-soluble drugs (including anticancer drugs) in the ASD formulation was mainly passive diffusion where only molecularly dissolved API was considerably absorbed by the intestinal epithelium. Furthermore, the permeability could only be enhanced by high concentrations of dispersed API. Therefore, drug absorption from dissolved ASD appears to be primarily driven by passive diffusion, which could be augmented by increasing the concentration of molecularly dissolved API, with the solubility of the amorphous drug being the limiting factor. Improving the bioavailability of poorly water-soluble anticancer drugs could also enhance their pharmacokinetics, efficacy, and safety. The speculated mechanism of bioavailability improvement from amorphous anticancer drugs in amorphous solid dispersion is summarized in Figure 5.

## 10. Conclusions

In conclusion, the oral dosage form was the most preferred route for delivering anticancer drugs. However, a significant drawback was that approximately 65% of these drugs exhibited poor water solubility. This limitation resulted in insufficient bioavailability, ultimately leading to suboptimal therapeutic outcomes with reduced efficacy and increased toxicity. The results of this study showed that ASD formulations offered a valuable solution, as they does not only improve the oral bioavailability but also enhance pharmacokinetics, thereby improving both efficacy and safety. Additionally, fundamental insight into the potential of ASD as a promising formulation for improving the aqueous solubility of poorly water-soluble anticancer drugs was provided.

## Figures and Tables

**Table 1:** List of anticancer drugs which have poor water solubility.

No.	Anticancer Drug	Structure	Anticancer Activity	Water Solubility	References
1.	Abiraterone acetate	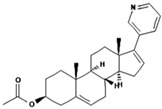	Inhibition of the CYP17A1 enzyme involved in androgen production in the body.	0.00305 mg/mL.	[26,27]
2.	Axitinib	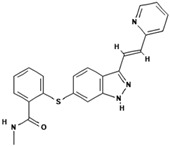	Inhibits vascular endothelial growth factor receptor (VEGFR), tyrosine kinase receptor.	Over 0.0002 mg/mL in aqueous media with pH ranging from 1.1 to 7.8. Solubility increases with decreasing pH.	[28,29]
3.	Bosutinib monohydrate	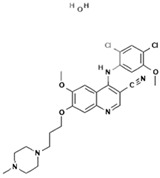	Inhibition of the protein kinase enzyme BCR-ABL, which plays an important role in cell development and proliferation.	Highly soluble at pH ≤ 5 (2 mg/mL) and the solubility reduces when pH increase (>5).	[30,31]
4.	Cabozantinib	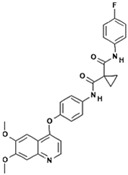	Inhibition of MET, RET, and angiogenesis pathways.	Practically insoluble in pH > 4.	[32,33]
5.	Ceritinib	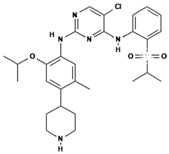	Binds to the ALK kinase tyrosine domain and prevents phosphorylation of downstream substrates, inhibiting signaling pathways that promote cancer cell growth.	Practically insoluble in water (0.02 mg/mL). pH-dependent solubility and pH increases become sparingly soluble with a solubility of 11 mg/mL at pH = 1 and 0.0002 mg/mL at pH = 6.8.	[34,35,36]
6.	Dabrafenib	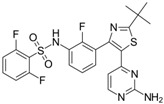	Inhibits abnormally activated BRAF proteins, which are responsible for cancer cell growth and proliferation.	Very slightly soluble at pH 1 and practically insoluble above pH 4.	[37,38]
7.	Erlotinib	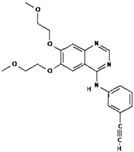	Inhibits epidermal growth factor receptor (EGFR) activity on cancer cells.	0.01402 mg/mL at 25 °C.	[39,40]
8.	Lapatinib	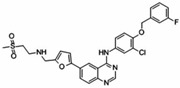	Inhibits HER2 and EGFR activity.	0.007 mg/mL at 25 °C.	[41,42]
9.	Midostaurin	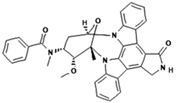	Inhibits overactivity of FLT3 protein involved in cancer cell growth and proliferation.	<1 mg/mL (very slightly soluble).	[43,44]
10.	Neratinib	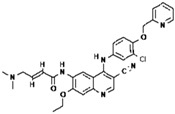	Inhibits HER2 overactivity involved in cancer cell growth and proliferation.	Sparingly soluble at pH 1.2 (32.90 mg/mL) and insoluble at pH ≥ 5.0 (0.08 mg/mL or less).	[45,46]
11.	Nilotinib	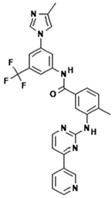	Inhibits the overactivity of BCR-ABL proteins involved in cancer cell growth and proliferation.	2.4 × 10^−5^ mg/mL at 25 °C.	[47,48]
12.	Nintedanib	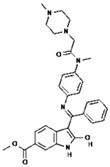	Inhibition of three receptor tyrosine kinases involved in angiogenesis, namely, platelet-derived growth factor (PDGF)-type receptor, vascular endothelial growth factor (VEGF)-type receptor, and fibroblast growth factor (FGF)-type receptor.	0.00966 mg/mL at 25 °C.	[49,50]
13.	Pazopanib	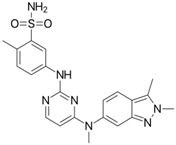	Inhibits several receptor tyrosine kinases, including VEGFR, PDGFR, FGFR, KIT, and RET. One of the main targets of pazopanib is VEGFR.	0.0033 mg/mL at 25 °C.	[51,52]
14.	Sonidegib	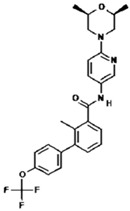	Inhibits the Hedgehog pathway, which is a signaling pathway involved in cell growth and differentiation.	Practically insoluble.	[53,54]
15.	Trametinib	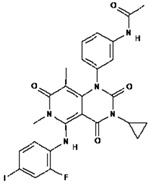	Inhibiting the MEK enzyme, trametinib interferes with the RAS/RAF/MEK/ERK pathway and stops cancer cell proliferation.	Practically insoluble.	[55,56]
16.	Vemurafenib	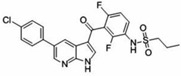	Inhibition of pathologically activated BRAF enzyme activity in melanoma with BRAF V600E mutation.	Practically insoluble (<0.0001 mg/mL).	[57,58]
17.	Regorafenib	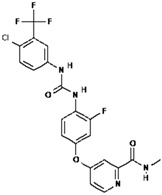	Inhibits specific enzymes involved in cell growth pathways and angiogenesis (formation of new blood vessels). Protein kinases involved in cancer cell growth pathways such as VEGFR, PDGFR, FGFR, KIT, and RET.	Practically insoluble.	[59,60]
18.	Everolimus	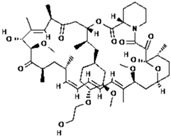	Inhibition of target of rapamycin (mTOR) in cancer cells.	Practically insoluble (<0.1 mg/mL).	[61,62]
19.	Venetoclax	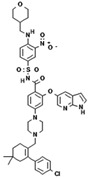	Inhibition of B-cell lymphoma anti-apoptotic protein 2 (BCL-2).	Practically insoluble.	[63,64]
20.	Olaparib	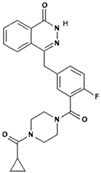	Inhibition of the PARP enzyme, which plays a role in repairing DNA damage.	Practically insoluble 0.1 mg/mL.	[65,66]

**Table 2:** List of some recent studies of poorly water-soluble drugs employed by the ASD system.

Active Compound	Polymer	Method	Results	References
Itraconazole	Polyvinylpyrrolidone vinyl acetate copolymer 88 and Hydroxy propyl methylcellulose acetate succinate	Solvent evaporation	HPMCAS showed good storage stability at an extended RH of more than 60% compared with PVPVA	[72]
Glibenclamide	Hypromellose acetate succinate	Anti-solvent addition method	Improved dissolution leads to the formation of glibenclamide-rich amorphous droplets	[73]
Atorvastatin	Pluronic F127 and Pluronic F68	Fusion method	Improved solubility and bioavailability compared to plain atorvastatin	[74]
Darunavir	HPMC/PVP	Coaxial electrospraying	Increase in drug-loading capacity and effect of gastro resistance on the molecule	[75]
Nobiletin	Methyl hesperidin mixture	Hot melt extrusion	Increased dissolution rate up to 7.5 times, permeability increases, and stable for up to 6 months under accelerated stability test conditions	[76]
Carvedilol	Β-cyclodextrin and hydroxypropyl-β-cyclodextrin	Complexation and kneading technique	A stable complex is formed and the dissolution rate increases in the range of pH 6.8 and 7.4	[77]
Fenofibrate	PVP K30, HPMC E6, HPMC E15	Hot melt extrusion	Significantly higher dissolution than the pure drug	[78]
Griseofulvin	Polyvinylpyrrolidone vinyl acetate polymer	Freeze drying	Significantly increased dissolution rate and oral absorption	[79]
Aripiprazole	Kollidon 12 PF	Hot melt extrusion	Increased dissolution compared to ordinary active substances and the use of acidifier increases bioavailability compared to not using it	[80]
Nevirapine	HPMCAS, hydroxypropyl methylcellulose phthalate, and Eudragit L100-55	Hot melt extrusion	Shows good dissolution because it is made with enteric polymers that are not affected by gastric pH	[81]
Indomethacin	PVP K25	Spray drying	Increased solubility, and improve physical stability	[82]
Ezetimibe	PVP K30	Solvent Method	Improved oral bioavailability	[83]
Carbamazepine	Methylcellulose	Hot melt extrusion	Increased dissolution compared to crystalline forms	[84]
Oridonin	PVP K17	Gas anti-solvent technique	Increased oral bioavailability	[85]
Telmisartan	PVP K30	Spray drying	Increased dissolution rate (around 3-fold) due to the optimum pH-modulated formulation	[86]
Itraconazole	PVP Vinyl Acetate	Electrospinning method	Improved dissolution rate and dissolution properties	[87]
Valsartan	HPMC	Spray drying	Increased solubility, dissolution, and bioavailability	[88]
Docetaxel and Paclitaxel	PVP K30	Spray drying	Increased solubility	[89]
Enzalutamide	HPMC and PVP-VA	Solvent evaporation	Increased dissolution, slower precipitation into aggregates of amorphous, increased bioavailability (in rats)	[90]
Nilotinib	Soluplus^®^	Spray drying	Increased solubility, increased bioavailability	[91]

**Table 3:** Examples of polymers commonly used employed in the formulation of ASD systems.

Polymers	Polymer Types	Molecular Weight (Da)	Transition Glass Temperature (°C)	Study	Reference
Povidone K25	Vinyl and its derivatives	30	153	PVP K25 can improve the stability of amorphous drugs	[115,116,117]
Povidone K17		10	140	PVP K17 showed better results than PVP K90 in terms of solubility	[85,116,118]
Povidone K30		50	160	PVP K30 has the most optimal crystallization inhibition activity than other PVPs that have lower or higher molecular weights	[116,119,120,121]
Carbomer	Polyacrylates and methacrylates	7 × 10^5^–4 × 10^9^	100–105	The addition of carbomer leads to prolonged dissolution	[122]
Eudragit^®^ EPO		135	52	At low pH, EPO improved the solubility of crystalline and amorphous forms of the drug but did not inhibit crystallization	[116,123,124]
Eudragit^®^ RL PO		32	63	Eudragit^®^ RL PO combined with hot melt extrusion method can solve the problems of bitter taste and poor solubility	[116,125,126]
Hydroxypropylmethylcellulose (HPMC)	Cellulose and its derivatives	10,000–1,500,000	170–180	The presence of HPMC acts as crystallization inhibitor by increasing supersaturation degree of drug	[127,128]
Hypromellose acetate succinate (HPMCAS)		55,000–90,000	113 ± 2	HPMCAS stabilized supersaturated drug solution, increases dissolution, and also acts as a precipitation inhibitor.	[129,130]
Methyl cellulose		10,000–220,000	175		[116,131]
Soluplus^®^	Other miscellaneous	90,000–140,000	~70	Soluplus^®^ enhances bioavailability by increase solubility based on the results of in vitro dissolution test	[84,132]
Kollicoat^®^ IR		~45,000	45	Kollicoat^®^ IR improves water penetration and wettability of drugs and leads to improved dissolution rate	[116,133,134]
Chitosan		10,000–1,000,000	203	Chitosan can increase the rate of dissolution, accompanied by a decrease in crystallinity and size of the drug which can increase dissolution	[135,136]

**Table 4:** ASD system of poorly water-soluble anticancer drugs.

Drug	Polymer	Interaction	Results	Reference
Total biflavonoid extract from *Selaginella deoderleinii* (TBESD)	PVP K30	FTIR: There was a shift in the C=O peak of PVP K-30 in TBESD-ASD from 1662.37 cm^−1^ to 1657.56 cm^−1^, indicating a hydrogen bond interaction between PVP K-30 and biflavonoids in TBESD, which indicates the hydrogen bonding interaction between PVP K-30 and biflavonoids in TBESD.	The results of the in vivo assay were that oral administration of TBESD-ASD to xenograft tumor-bearing mice resulted in a significant reduction in microvascular density and tumor size.	[248]
Low-dose paclitaxel	PVP K30 + sodium lauryl sulfate (SLS)	FTIR: There is a peak in the spectrum of the C=O group at 1700 cm^−1^ where the sharp double peak of paclitaxel di-hydrate turns into a single blunt peak, indicating the interaction of hydrogen groups.	Paclitaxel solid dispersion formulation (ModraPac100 capsules) has (almost) the same pharmacokinetic profile as the low-dose paclitaxel premix solution.	[187]
Paclitaxel (PTX)	PVP/VA S-630 TPGS	FTIR: Changes The peak spectra of amide I (-CO-NH-) in the SD4 and SD9 formulations significantly changed from 1645.2 cm^−1^ in paclitaxel to 1677.8 cm^−1^ (SD4) and 1660.8 cm^−1^ (SD9), indicating hydrogen bond interactions.	SD4 and SD9 formulations have significantly increased anticancer effects on breast cancer cells compared to PTX alone.	[249]
RA-XII isolated from R. *yunnanensis*	PVP K17 PVP K90 PVP K30	FTIR: There is a shift in the spectrum of O-H and C=O groups, which indicates the hydrogen interaction between RA-XII and PVP.	The ASD formulation of RA-XII inhibited the proliferation of RKO cells better than free RA-XII. ASD formulation of RA- XII has a stronger therapeutic effect on colon cancer cells.	[250]
IIIM-290	PEG-PPG-PEG PVP K-30 Gom Xanthan	FTIR: The presence of peak broadening in the hydroxyl group (O-H) region indicates the hydrogen bonding interaction of drug–polymer.	Solid dispersion injection in mice with Ehrlich ascites carcinoma resulted in a 1.9-fold increase in plasma exposure, resulting in 31–43% tumor inhibition.	[251]

## Data Availability

Not applicable.
